# Real-World Oncological Outcomes of Nivolumab Plus Ipilimumab in Advanced or Metastatic Renal Cell Carcinoma: A Multicenter, Retrospective Cohort Study in Japan

**DOI:** 10.3390/curroncol31120583

**Published:** 2024-12-11

**Authors:** Tomoki Taniguchi, Koji Iinuma, Kei Kawada, Takashi Ishida, Kimiaki Takagi, Masayuki Tomioka, Makoto Kawase, Kota Kawase, Keita Nakane, Yuki Tobisawa, Takuya Koie

**Affiliations:** 1 Department of Urology, Graduate School of Medicine, Gifu University, 1-1 Yanagido, Gifu 501-1194, Japan; taniguchi.tomoki.a8@f.gifu-u.ac.jp (T.T.); iinuma.koji.s0@f.gifu-u.ac.jp (K.I.); tomioka.masayuki.e4@f.gifu-u.ac.jp (M.T.); kawase.makoto.g5@f.gifu-u.ac.jp (M.K.); kawase.kota.b5@f.gifu-u.ac.jp (K.K.); nakane.keita.k2@f.gifu-u.ac.jp (K.N.); tobisawa.yuki.a7@f.gifu-u.ac.jp (Y.T.); 2 Department of Urology, Ogaki Municipal Hospital, 4-86 Minaminokawa-cho, Ogaki 503-8502, Japan; 3 Department of Urology, Gifu Prefectural General Medical Center, 4-6-1 Noisiki, Gifu 500-8717, Japan; keinedvedon@yahoo.co.jp; 4 Department of Urology, Gifu Municipal Hospital, 7-1 Kashima-cho, Gifu 500-8513, Japan; justaskaxis@gmail.com; 5 Department of Urology, Daiyukai Hospital, 1-9-9 Sakura, Ichinomiya 491-8551, Japan; ktakagi@daiyukai.or.jp

**Keywords:** renal cell carcinoma, nivolumab, ipilimumab, Japanese cohort

## Abstract

A combination of nivolumab and ipilimumab (NIVO + IPI) is the only approved combination of two immune checkpoint inhibitors for metastatic or advanced renal cell carcinoma (mRCC). Inadequate evidence of treatment with NIVO + IPI has been reported in Japanese cohorts. We evaluated the clinical efficacy of NIVO + IPI treatment. Patients with mRCC who received NIVO + IPI at nine Japanese facilities between August 2018 and March 2023 were enrolled in this study. The primary endpoint in this study was the assessment of oncological outcomes in patients with mRCC who received NIVO + IPI. Eighty-four patients with mRCC were enrolled. The median follow-up period was 18.3 months, and median progression-free and overall survival were 13.3 and 50.9 months, respectively. The objective response rate was 47.6%, and the disease control rate was 78.6%. To our knowledge, this is the largest study that evaluates Japanese patients with mRCC receiving NIVO + IPI treatment. In this study, the real-world oncological outcomes after NIVO + IPI treatment were comparable to those in the CheckMate 214 study.

## 1. Introduction

Renal cell carcinoma (RCC) is the 14th most common malignant neoplasm worldwide, and approximately 430,000 patients were likely to be newly diagnosed with RCC in 2022 [1,2]. According to the National Cancer Center database, approximately 21,000 patients were diagnosed with RCC in 2019 [3]. Interferon-alpha or interleukin-2 has previously been used as a first-line therapy for metastatic or progressive RCC (mRCC), as this malignant tumor is highly resistant to cytotoxic chemotherapy [4]. Response rates to these therapies are relatively low, with a median overall survival of approximately 12 months [4]. Subsequently, molecularly targeted therapies, such as tyrosine kinase inhibitors (TKIs), were introduced for the treatment of mRCC, and their oncological outcomes were significantly improved compared to those of previous therapies [4,5,6]. The International Metastatic Renal Cell Carcinoma Database Consortium (IMDC) risk classification is a widely validated model for predicting the prognosis of mRCC and is divided into three groups based on clinical and laboratory data: favorable, intermediate, and poor risks [7]. The median overall survival (OS) with molecularly targeted therapy has been reported to be 43.2 months in patients with favorable-risk, 22.5 months in intermediate-risk, and 7.8 months in poor-risk mRCC [7].

More recently, combination therapies with immune checkpoint inhibitors (ICIs) and TKIs have been used as first-line treatments for mRCC, further improving oncological outcomes, including OS and progression-free survival (PFS) in patients with mRCC [8,9,10,11,12,13]. Among these, nivolumab plus ipilimumab (NIVO + IPI) is the only ICI combination therapy for mRCC. According to the Checkmate 214 study, a randomized, open-label phase III trial, patients treated with NIVO + IPI had a significantly longer OS and higher objective response rate (ORR) than those treated with sunitinib (SUN) [9]. The Checkmate 214 trial enrolled 1096 patients with mRCC, of whom only 60 were Japanese [14]. Although the efficacy of NIVO + IPI treatment for Japanese patients in a mRCC cohort and the efficacy of second-line treatment after NIVO + IPI have been reported, the relatively short follow-up period and small number of eligible patients are considered limitations for these studies [15,16].

Therefore, we evaluated the real-world efficacy of NIVO + IPI therapy in patients with mRCC and investigated the efficacy of TKIs as second-line therapies.

## 2. Materials and Methods

### 2.1. Patients

We retrospectively reviewed the clinical records of patients with mRCC who were classified as intermediate or poor risk according to the IMDC risk classification and who received NIVO + IPI between August 2018 and March 2023 at nine centers in Japan. Patients aged < 20 years, those who refused to participate in the study, and those with missing data were excluded from the analysis. Patient characteristics, such as age, sex, height, weight, Eastern Cooperative Oncology Group performance status (ECOG-PS), IMDC risk classification, pathological features, whether the patient underwent surgery, and metastatic sites were collected.

### 2.2. Treatment

Prior to September 2018, the patients received 3 mg/kg of NIVO and 1 mg/kg of IPI every three weeks, whereas after October 2018, they received 240 mg of NIVO and 1 mg/kg of IPI. After four courses of NIVO + IPI, the patients received 240 mg of NIVO every two weeks or 480 mg of NIVO every four weeks. This treatment was continued until disease progression, unacceptable toxicity as a treatment-related adverse event, or other reasons for discontinuation.

### 2.3. Evaluation

According to the American Joint Committee on Cancer Staging Manual, tumor stage of each patient was assessed using computed tomography (CT) [17]. The enrolled patients in the study underwent CT every 1–3 months during or after treatment with NIVO + IPI. The overall response was assessed with the Response Evaluation Criteria in Solid Tumors guidelines (version 1.1) [18] as complete response (CR), partial response (PR), stable disease (SD), or disease progression (PD). The ORR was defined as the proportion of patients whose best response (BOR) was CR or PR. The disease control rate (DCR) was defined as the proportion of patients whose BOR was CR, PR, or SD.

### 2.4. Statistical Analysis

The primary endpoints of this study were oncological endpoints, including OS and PFS. The secondary endpoints were BOR, ORR, and DCR as measures of disease control. All statistical analyses in this study were conducted with EZ-R software version 1.65 (Saitama Medical Center, Jichi Medical University, Saitama, Japan) [19]. Patient characteristics were described using medians and interquartile ranges for continuous variables and percentages for categorical variables. Continuous variables were analyzed with the Mann–Whitney U test, and categorical variables were analyzed using the chi-squared test. OS was defined as the time from initiation of NIVO + IPI therapy to the date of death from cancer or other causes. Progression-free survival (PFS) was defined as the time from the date of the first NIVO + IPI administration to the date of disease progression or death. Duration of response (DOR) was defined as the time from the date of CR or PR to the date of PD. OS and PFS were described using the Kaplan–Meier method. Statistical significance was set at *p* < 0.05 for all comparisons.

## 3. Results

### 3.1. Patient Characteristics

Eighty-four patients were enrolled in the study with a median follow-up period of 18.3 months (interquartile range [IQR], 9.4–34.9). Patient characteristics and clinical data according to the risk in the IMDC classification are shown in Table 1. The median age of all the patients was 68.0 years, and median body mass index was 22.6 kg/m^2^ (IQR, 20.7–24.8 kg/m). Sixty patients (71.4%) were diagnosed with clear cell RCC, and the most common site of metastasis was the lungs (63.1%). Thirty-three patients (70.2%) with intermediate-risk RCC and eighteen (48.6%) with poor-risk RCC had undergone nephrectomy prior to NIVO + IPI (*p* = 0.074).

### 3.2. Oncologic Outcomes

**Figure 1:**
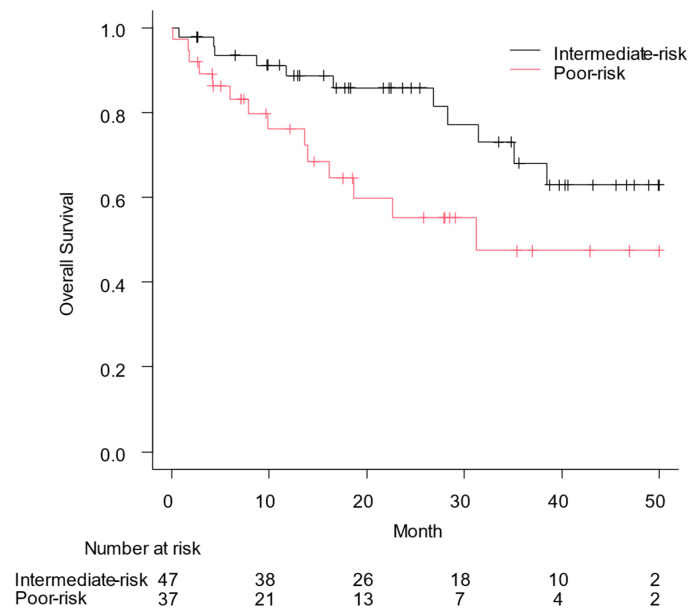
Overall survival (OS) from the date of the first administration of nivolumab plus ipilimumab to the date of all-cause mortality was assessed using the Kaplan–Meier curve. Median OS was not reached in patients with intermediate-risk renal cell carcinoma and 31.2 months in those with poor-risk renal cell carcinoma (*p* = 0.028).

**Figure 2:**
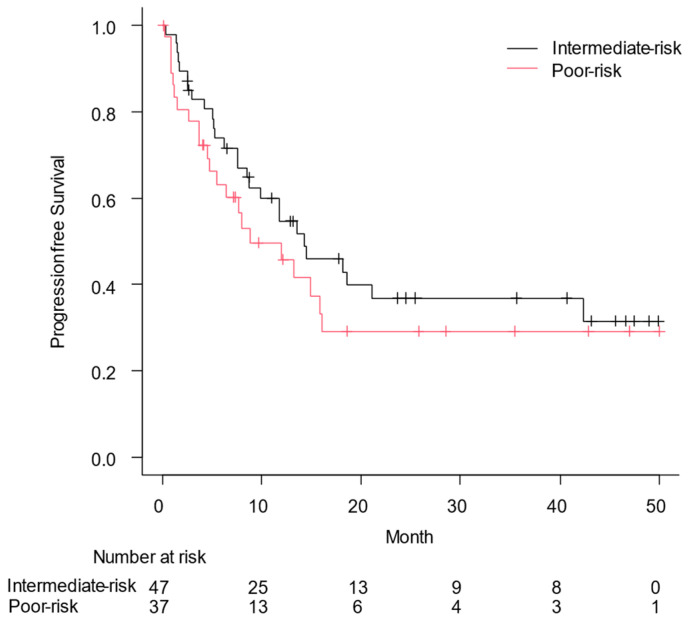
Progression-free survival (PFS) from the date of the first administration of nivolumab plus ipilimumab to the date of disease progression or all-cause death was assessed using the Kaplan–Meier curve. Median PFS was 14.3 months in patients with intermediate risk of renal cell carcinoma and 8.9 months in those with poor risk (*p* = 0.34).

Figure 1 shows the OS based on the IMDC risk classification; median OS was not reached in patients with intermediate-risk RCC and it was 31.2 months in those with poor-risk RCC (*p* = 0.028). The PFS for the enrolled patients is shown in Figure 2, with a PFS of 14.3 months according to the IMDC risk category for patients with intermediate-risk RCC and 8.9 months for those with poor-risk RCC (*p* = 0.34). In particular, the median OS and PFS for patients with PS3/4 were 51.0 (95% confidence interval [CI], 0.1: not applicable) and 5.2 months (95% CI, 1.5–13.3), respectively.

Table 2 shows the treatment effects of IMDC risk classification. The ORR and DCR for the entire cohort were 47.6% and 78.6%, respectively. The median DOR was 41.0 months for enrolled patients. There was no difference between patients with intermediate- and poor-risk RCC in terms of DOR (*p* = 0.966). A total of 55 patients experienced any grade of immune-related adverse events (irAEs) and 37 required steroids. The irAEs included a rash in 19 (22.6%), adrenal insufficiency in 15 (17.9%), hypothyroidism in 12 (14.3%), liver dysfunction in 10 (11.9%), interstitial pneumonia in 8 (9.5%) and colitis in 7 (8.3%). At the end of the study, 13 patients were still receiving NIVO + IPI. In addition, 34 patients (40.5%) discontinued NIVO + IPI due to PD and 27 (32.1%) due to irAE. Of the patients who discontinued treatment due to irAE or patient preference, 16 survived without disease progression.

### 3.3. Subsequent Therapy After NIVO + IPI Treatment

Table 3 lists the types of second-line treatments after NIVO + IPI. Forty-one patients (48.8%) received second-line therapy, all of whom were treated with TKIs. After NIVO + IPI treatment resulted in PD, eight patients failed to receive second-line treatment due to disease progression.

Table 4 shows the BOR of the second-line therapies. The ORR and DCR for all the patients were 56.1% and 73.2%, respectively, with a trend towards more successful TKIs in patients with intermediate risk, although the difference was not statistically significant.

## 4. Discussion

The Japanese overall mortality rate of malignant renal tumors has been increasing, whereas the age-standardized mortality rate has been decreasing since 2016 [20]. In contrast, the incidence of RCC increased by approximately 1% per year between 2015 and 2019, whereas its mortality rate decreased by approximately 2% per year between 2016 and 2020 in the United States [2]. It was speculated that these trends are due to improvements in treatment in addition to an increase in the early detection rate of RCC [2].

The number of pharmacological treatment options for RCC has increased in recent years, and various ICI-based combination therapies are now available, depending on the disease progression status, number and location of metastases, and patient’s ECOG-PS. The CheckMate 214 trial was a randomized, open-label, phase 3 study designed to evaluate the oncological outcomes of NIVO + IPI versus SUN monotherapy. OS in the NIVO + IPI arm was superior to that in the SUN arm when the IMDC risk classification was intermediate- or poor-risk RCC [9]. The study was initially reported as an interim analysis, with a median follow-up of 25.2 months [9]. Subsequently, combined ICI and TKI regimens were introduced for patients with RCC. A randomized phase III, open-label study conducted by the KEYNOTE-426 trial enrolled 861 patients with mRCC who received pembrolizumab plus axitinib (PEM + AXI) or SUN, with a median follow-up of 12.8 months [10]. The one-year OS rate was 89.9% for patients treated with PEM + AXI and 78.3% for those treated with SUN (*p* < 0.001) [10]. Median PFS was 15.1 months in the PEM + AXI group and 11.1 months in the SUN group (*p* < 0.001) [10]. A randomized phase III JAVELIN Renal 101 trial in 886 patients with mRCC found that avelumab plus AXI (AVE + AXI) prolonged PFS compared to SUN (13.8 months vs. 8.4 months) (*p* < 0.001) [11]. However, the study found no statistically significant difference in OS between AVE + AXI and SUN (one-sided *p* = 0.012) [11]. A phase 3, open-label, randomized CheckMate 9ER trial comparing the treatment efficacy of NIVO + cabozantinib (CABO) and SUN in patients with mRCC enrolled 651 patients with a median follow-up period of 18.1 months [12]. The median PFS was 16.6 months for NIVO + CABO and 8.3 months for SUN (*p* < 0.001) [12]. The one-year OS rate was 85.7% for NIVO + CABO and 75.6% for SUN (*p* = 0.001) [12]. In the CLEAR trial conducted to compare the efficacy of PEM plus lenvatinib (PEM + LEN) versus SUN in patients with mRCC, PFS was significantly prolonged in patients receiving PEM + LEN compared to those receiving SUN (*p* < 0.001) [13]. Additionally, the two-year OS rate was significantly prolonged (79.2% in the PEM + LEN group and 70.4% in the SUN group, *p* = 0.005) [13].

Extended follow-up results have reported the therapeutic efficacy of NIVO + IPI therapy in mRCC [21,22,23]. Results from the recent CheckMate 214 trial, which extended the median follow-up period to 99.1 months, showed that in patients with intermediate- or poor-risk RCC according to the IMDC risk classification, median OS was 52.7 months for NIVO + IPI versus 37.8 months for SUN (hazard ratio [HR], 0.72: 95% confidence interval [CI], 0.62–0.83) [23]. The median PFS was reported to be 12.4 months for NIVO + IPI compared to 12.3 months for SUN, and the 90-month PFS probability was 22.8% compared to 10.8% [23]. The ORR was 42.4% for NIVO + IPI and 27.5% for SUN therapy, with a CR rate of 12.0% versus 3.5% for each treatment group [23]. Similarly, the median DOR was 82.8 and 19.8 months in the NIVO + IPI and SUN groups, respectively, with a higher response rate at 90 months in the NIVO + IPI group than that in the SUN group (50% vs. 23%) [23]. Treatment with NIVO + IPI has been shown to be durable compared to other treatment regimens, and 41 of 66 patients (62.1%) who achieved CR with NIVO + IPI did not receive a subsequent treatment by the cut-off date and were followed-up without treatment [23]. This suggests that patients who respond to NIVO + IPI treatment may benefit from a long-lasting therapeutic effect.

The different ORRs and DORs for each of the therapies in the phase 3 trials to date should also be noted. The ORRs of the KEYNOTE-426, JAVELIN Renal 101, CheckMate 9ER, and CLEAR trials were 59.3%, 51.4%, 55.7%, and 71.0%, respectively, while the SD rates were 10.9%, 11.5%, 5.6%, and 5.4% in each arm, respectively [10,11,12,13]. Among the ICI + TKI combinations, PEN + LEN showed relatively low PD rates and a high ORR. In contrast, the ORR in the CheckMate 214 trial was 41.0%, with a trend towards a lower BOR than that of other ICI + TKI regimens [9]. The ICI + TKI regimen has a relatively lower PD rate than the NIVO + IPI regimen, which may be useful for patients who have symptoms or rapid disease progression requiring relatively early tumor reduction. The PFS and ORR of the AVE + AXI or PEM + LEN groups decreased with increasing IMDC risk factors, similar to those in the SUN group [10,13]. However, NIVO + IPI showed consistent ORR efficacy, regardless of the number of risk factors compared to SUN, although no CR was observed in mRCC patients with 4–6 IMDC risk factors [24]. Therefore, it has been suggested that NIBO + IPI may provide long-term durability compared with ICI + TKI treatment. Although the follow-up period in this study was relatively short, the median time to DOR was 41 months, which indicates a relatively long-term response.

In a Japanese cohort, the CheckMate 214 trial enrolled 38 patients receiving NIVO + IPI and 34 receiving SUN [14]. Demographic and baseline characteristics of the Japanese patients were similar to those of all patients enrolled in the CheckMate 214 trial [14]. The median OS in this cohort was not reached with NIVO + IPI and 33.4 months with SUN [14]. The twenty-four-month OS rate was 84% in the NIVO + IPI group and 76% in the SUN group, showing a trend for Japanese patients treated with NIVO + IPI to benefit from treatment later in the course of treatment compared to patients treated with SUN (*p* = 0.267) [14]. Although few Japanese patients were enrolled in the randomized phase 3 CheckMate 214 trial, there are currently several reports on the oncological outcomes of NIVO + IPI in Japanese patients with mRCC [15,25,26]. In the J-Cardinal study, a Japanese retrospective observational study enrolled 45 patients with mRCC, and the median overall follow-up period was 24 months [25]. The median OS was not achieved, and the median PFS was 17.8 months, with 24-month OS and PFS rates of 59.1% and 41.6%, respectively, and an ORR of 41.5% for all enrolled patients [25]. The present study, which enrolled 84 Japanese patients with mRCC, was similar to previous reports. In real-world clinical practice, NIVO + IPI may be administered to patients with reduced PS due to tumor-related symptoms. The results of this study suggest that NIVO + IPI is an effective therapy even in cohorts that include patients with worse PS than those enrolled in the clinical trial. The CR rate in patients with intermediate-risk RCC in this study was also 21.3%. Additionally, relatively more patients with intermediate-risk RCC had undergone nephrectomy prior to treatment with NIVO + IPI than those with low-risk RCC. This may have influenced the oncological outcomes. The number of patients with brain metastases enrolled in this study was slightly higher than in other studies [14,26]. In contrast, the ORR for patients with brain metastases was 80%, indicating a relatively favorable response to tumor reduction. However, the small number of patients may limit the therapeutic significance of NIVO+IPI in patients with brain metastases.

Several guidelines recommend treatment with TKIs as second-line or subsequent sequential therapy after combination therapy, including ICIs, for mRCC [3,27]. In a multicenter phase II trial, 40 patients with mRCC that had progressed after ICI treatment received at least one course of AXI as second-line therapy and its efficacy was evaluated [28]. With a median follow-up of 8.7 months, the median PFS was 8.8 months with an ORR of 45% [28]. Of the 18 patients who responded to AXI, 12 (67%) showed a sustained response for >12 months [28]. The most common grade 3 adverse events were fatigue (8%), hypertension (60%), and hand–foot syndrome (8%) [28]. Although eight patients (20%) experienced serious adverse events that were at least likely to be related to AXI treatment, no patients discontinued treatment due to an adverse event, and there were no deaths [28]. The phase 2 BREAKPOINT trial was conducted as a prospective, single-arm, multicenter trial that enrolled 31 patients who received CABO [29]. At a median follow-up of 11.9 months, median OS and PFS were 13.8 and 8.3 months, respectively [29]. The ORR was 37.9%, and 13 patients achieved stable disease [29]. A randomized, placebo-controlled, double-blind, pivotal CANTANA trial enrolled patients with mRCC who had received prior therapy, including NIVO + IPI [30]. The enrolled patients were randomized to receive CABO plus telaglenastat or CABO plus a placebo [3]. The results of this study showed that CABO treatment resulted in a median PFS of approximately nine months and an ORR of approximately 30%, although no additive effect of telaglenastat was observed [3]. Based on the results of these studies and the fact that DCR was observed in 73.2% of the patients in this study, TKIs could be expected to have some efficacy as a second-line therapy following NIVO + IPI. TKIs may be effective as second-line therapy for patients with PD after receiving NIVO + IPI. Therefore, we believe there is a need to switch to second-line treatment with TKIs as soon as possible in patients with PD after NIVO + IPI treatment.

However, this study has several limitations. First, this was a retrospective, multicenter cohort study and was subject to potential bias. Treatment decisions were based on institutional criteria, which may have resulted in the selection of other treatment regimens for patients receiving combined ICI therapy. Although patients enrolled in CheckMate 214 were treated with NIVO + IPI for 2 years, this cohort includes patients who continued treatment after 2 years. Therefore, it is suggested that there may be bias in the interpretation of the results obtained in this study. Second, the number of patients enrolled was small, and the median follow-up period was relatively short. Therefore, long-term results in real-world settings are warranted. Third, the decision of which drug to use as a second-line treatment also depended on the institution, physician, and patient preferences. This may have introduced selection bias. As some patients may benefit from TKIs as a second-line therapy, the appropriate timing for switching to second-line therapy needs to be considered.

## 5. Conclusions

In this study, the oncological outcomes with NIVO + IPI in Japanese patients with mRCC in real-world practice were similar to those in the randomized phase II Checkmate 214 trial, and TKI administered as second-line therapy also achieved reasonable disease control. Therefore, a large prospective study of Japanese patients with mRCC is warranted.

## Figures and Tables

**Table 1:** Characteristics of patients in the two groups according to the risk categories of the IMDC.

	Intermediate Risk	Poor Risk	*p*
Number of patients (%)	47 (56.0)	37 (44.0)	
Age (year, median, IQR)	67.0 (58.5–72.0)	70.0 (65.0–74.0)	0.144
Sex (number, %)			0.056
Male	41 (87.2)	25 (67.6)	
Female	6 (12.8)	12 (32.4)	
Body mass index (median, IQR)	22.8 (21.1–24.7)	22.2 (20.3–25.3)	0.425
ECOG-PS (number, %)			0.188
0	29 (61.7)	16 (43.2)	
1	12 (25.5)	9 (24.3)	
2	4 (8.5)	6 (16.2)	
3	1 (2.1)	5 (13.5)	
4	1 (2.1)	1 (2.7)	
Pathology (number, %)			0.251
Clear cell RCC	37 (78.7)	23 (62.2)	
Papillary RCC	0 (0)	1 (2.7)	
Chromophobe RCC	1 (2.1)	0 (0)	
Unclassified	1 (2.1)	3 (8.1)	
No tissue diagnosis	8 (17.0)	10 (27.0)	
Sarcomatoid feature (number, %)	2 (4.3)	4 (10.8)	0.222
Metastatic sites (number, %)			
Lymph node	17 (36.2)	15 (40.5)	0.855
Lung	28 (59.6)	25 (67.6)	0.599
Liver	5 (10.6)	11 (29.7)	0.053
Bone	14 (29.8)	16 (43.2)	0.294
Brain metastasis	14 (29.8)	16 (43.2)	0.294
Follow-up period (months, median, IQR)	22.6 (12.1–38.6)	14.0 (5.9–28.0)	0.033

IMDC, International Metastatic RCC Database Consortium; IQR, interquartile range; ECOG-PS, Eastern Cooperative Oncology Group Performance Status; RCC, renal cell carcinoma.

**Table 2:** Response rates for nivolumab plus ipilimumab combination therapy in patients with mRCC according to the IMDC risk classification.

	Intermediate Risk	Poor Risk	*p*
Best objective response (number, %)			0.186
Complete response (CR)	10 (21.3)	2 (5.4)	
Partial response (PR)	12 (25.5)	16 (43.2)	
Stable disease (SD)	16 (34.0)	10 (27.0)	
Progression disease (PD)	8 (17.0)	8 (21.6)	
Unassessed	1 (2.1)	1 (2.1)	
Objective response rate (CR + PR) (number, %)	22 (46.8)	18 (48.6)	>0.999
Disease control rate (CR + PR + SD) (number, %)	38 (80.9)	28 (75.7)	0.760

IMDC, International Metastatic Renal Cell Carcinoma Database Consortium.

**Table 3:** Second-line treatment in two groups based on the IMDC risk classification.

Treatment (Number, %)	Intermediate Risk	Poor Risk	*p*
Axitinib	14 (58.3)	4 (23.5)	0.059
Cabozantinib	6 (25.0)	10 (58.8)	
Sunitinib	4 (16.7)	3 (17.6)	

IMDC, International Metastatic Renal Cell Carcinoma Database Consortium.

**Table 4:** Second-line response rate between two groups according to the IMDC risk classification.

	Intermediate Risk	Poor Risk	*p*
Best objective response (number, %)			0.206
Complete response (CR)	1 (4.2)	2 (11.8)	
Partial response (PR)	14 (58.3)	6 (35.3)	
Stable disease (SD)	5 (20.8)	2 (11.8)	
Progression disease (PD)	4 (16.7)	7 (41.2)	
Objective response rate (CR + PR) (number, %)	15 (62.5)	8 (47.1)	0.508
Disease control rate (CR + PR + SD) (number, %)	20 (83.3)	10 (58.8)	0.165

IMDC, International Metastatic Renal Cell Carcinoma Database Consortium.

## Data Availability

The data presented in this study are available on request from the corresponding author. The data are not publicly available due to privacy and ethical reasons.
